# Ergogenic effects of caffeine are mediated by myokines

**DOI:** 10.3389/fspor.2022.969623

**Published:** 2022-12-08

**Authors:** Shingo Takada, Yoshizuki Fumoto, Shintaro Kinugawa

**Affiliations:** ^1^Department of Lifelong Sport, School of Sports Education, Hokusho University, Ebetsu, Japan; ^2^Department of Molecular Biology, Hokkaido University Graduate School of Medicine, Sapporo, Japan; ^3^Department of Cardiovascular Medicine, Faculty of Medical Sciences, Kyushu University, Fukuoka, Japan; ^4^Division of Cardiovascular Medicine, Research Institute of Angiocardiology, Faculty of Medical Sciences, Kyushu University, Fukuoka, Japan

**Keywords:** myokine, exercise mimetic, mitochondria, ergogenic aid, calcium ion (Ca)^2+^, Ca^2+^-induced Ca^2+^ release, skeletal muscle, BDNF

## Abstract

Exercise has long been known to effectively improve and enhance skeletal muscle function and performance. The favorable effects of exercise on remote organs other than skeletal muscle are well known, but the underlying mechanism has remained elusive. Recent studies have indicated that skeletal muscle not only enables body movement, but also contributes to body homeostasis and the systemic stress response *via* the expression and/or secretion of cytokines (so-called myokines). Not only the induction of muscle contraction itself, but also changes in intracellular calcium concentration ([Ca^2+^]i) have been suggested to be involved in myokine production and secretion. Caffeine is widely known as a Ca^2+^ ionophore, which improves skeletal muscle function and exercise performance (i.e., an “ergogenic aid”). Interestingly, some studies reported that caffeine or an increase in [Ca^2+^]i enhances the expression and/or secretion of myokines. In this review, we discuss the association between caffeine as an ergogenic aid and myokine regulation.

## Introduction

**Figure 1:**
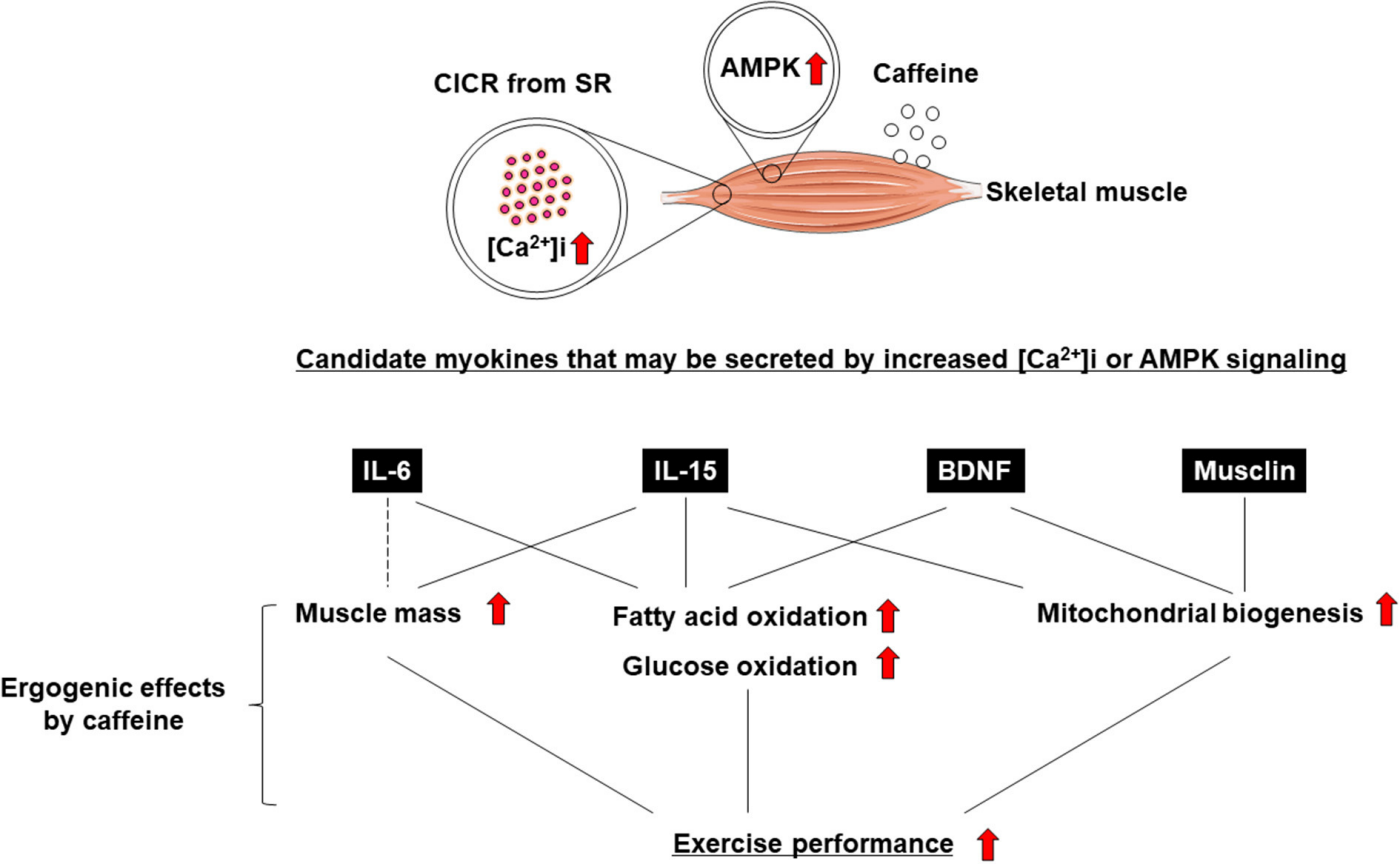
Ergogenic effects of caffeine are mediated by myokines.

Exercise training has been demonstrated to have positive effects on skeletal muscle function and systemic exercise performance (1, 2). The favorable effects of exercise on remote organs other than skeletal muscle are well known (3, 4), but the underlying mechanism remains unclear. Recent studies have indicated that skeletal muscle not only enables body movement, but also contributes to body homeostasis and the systemic stress response *via* the secretion of soluble proteins (3, 4). It has been suggested that these proteins, which are cytokines and other peptides that are produced, expressed, and secreted by muscle fibers, and exert paracrine, autocrine, or endocrine effects, should be classified as “myokines” (5). Not only the induction of muscle contraction itself, but also changes in intracellular calcium concentration ([Ca^2+^]i) have been suggested to be involved in myokine production and secretion (6–10) (Figure 1). It is also known that myokine secretion is promoted by the activation of 5′-AMP-activated protein kinase (AMPK) signaling (11, 12) (Figure 1).

Caffeine is widely known as a Ca^2+^ ionophore and/or AMPK activator that improves skeletal muscle function and exercise performance (i.e., an “ergogenic aid”) (13–16) (Figure 1). Some studies reported that caffeine, an increase in [Ca^2+^]i, and AMPK agonists enhance the secretion of myokines, leading to positive effects on skeletal muscle (e.g., increased fatty acid or glucose oxidation and mitochondrial biosynthesis) and exercise performance. (7–9, 11, 12, 17). In this review, we discuss the association between caffeine as an ergogenic aid and myokine regulation.

## Evidence of the ergogenic effects of caffeine

Short-term or long-term administration of caffeine has ergogenic effects, including enhancing fatty acid or glucose oxidation, mitochondrial biogenesis, and muscle hypertrophy signaling in cultured skeletal muscle cells and increasing skeletal muscle mass in rodents (18–20). Similarly, caffeine was identified as an ergogenic aid for exercise performance, including aerobic endurance, muscle strength, and muscle endurance in humans by meta-analyses (14). Thus, evidence of the ergogenic effects of caffeine on exercise performance is well-established (14, 15, 18–22).

## Ergogenic effects of caffeine are mediated by myokine secretion due to increased [Ca^2+^]i and/or AMPK activation

One mechanism of the above-mentioned ergogenic effects of caffeine involves calcium-induced calcium release from the sarcoplasmic reticulum, which increases [Ca^2+^]i (23–25). Increased [Ca^2+^]i is involved not only in protein expression and/or modification by enhancing the calcium signaling, including the AMPK pathway, but also in the extracellular secretion of proteins (6, 7, 11, 26, 27).

Myokine production is thought to occur in response to muscle contraction (5, 28), but the detailed mechanism remains unclear. Interestingly, it has been shown that myokine secretion during acute electrical stimulation depends more on intracellular calcium flux than on skeletal muscle contraction itself (9). Moreover, increased [Ca^2+^]i has the potential to promote myokine expression in skeletal muscle (7, 8, 29). On the other hand, caffeine is a well-known activator of AMPK (30, 31), and AMPK activation is involved in myokine regulation (11, 12). Next, we will focus on representative myokines that can be regulated by increasing [Ca^2+^]i and/or AMPK activation, which have recently been shown to be involved in the function of skeletal muscle and other organs, as well as exercise performance.

### Caffeine regulates the secretion of interleukin-6 as a myokine

Exercise was found to increase the levels of circulating and muscle interleukin (IL)-6, which is the most well-known myokine, in humans (5). Similarly, caffeine was found to increase circulating and skeletal muscle IL-6 protein levels in mice (17). A23187, another Ca^2+^ ionophore, also increased IL-6 mRNA expression in the skeletal muscle of mice and C2C12 myotubes (32). Moreover, it is well known that IL-6 promotes fatty acid and glucose oxidation in humans and in tissue culture (5, 33–35). Although the results are controversial, IL-6 also is involved in muscle hypertrophy and myogenesis (28, 36).

In general, mM levels of caffeine have been shown to promote Ca^2+^ release from the sarcoplasmic reticulum (SR) by acting directly on the ryanodine receptor (RyR) (6, 37). Ducreux et al. showed that upon activation of the RyR by the RyR agonist 4-chloro-m-cresol, myotubes released IL-6; this was dependent on *de novo* protein synthesis and was blocked by dantrolene (a substance that specifically closes calcium channels, thereby blocking calcium release from the SR) and cyclosporine (a substance that blocks calcium-dependent calcineurin activation by nuclear factor of activated T-cells) (6). Moreover, in an experiment in which caffeine was added to C2C12 skeletal muscle cultured cells, Fang et al. observed that mM-level caffeine secreted IL-6 in the culture supernatant (17). However, this report did not confirm whether caffeine administration affects [Ca^2+^]i. In addition, it has not been confirmed whether the caffeine-induced IL-6 secretion is suppressed by decreasing [Ca^2+^]i. On the other hand, in an experiment in which IL-6 was secreted by the contraction of C2C12 cultured skeletal muscle cells induced by electrical stimulation, it was reported that the increase in [Ca^2+^]i was more important than the contraction of the cells themselves (9). Therefore, an increase in [Ca^2+^]i is considered to be important for the secretion of IL-6. These results suggest that caffeine can regulate the secretion of IL-6 through an increase in [Ca^2+^]i. In addition, both physiological concentrations and μM levels of caffeine also act directly on skeletal muscle to bring about an ergogenic effect, and it is thought that the mobilization of intracellular calcium is also involved in this effect (38). Conversely, caffeine did not affect (i.e., did not induce) IL-6 vesicle secretion even after 70 min of intravenous administration to mice at the highest possible dose (85 mg/kg) (11). In this study, confocal imaging was used to visualize the endogenous IL-6 protein in glycolytic fast fibers of the tibialis anterior muscle of mice. Moreover, 2 h of incubation of either the extensor digitorum longus (EDL) or soleus muscle from mice with the Ca^2+^ ionophore ionomycin in the medium did not significantly increase IL-6 levels (39). However, ionomycin stimulation showed a tendency of an increase in IL-6 release from skeletal muscle, particularly from soleus muscle. On the other hand, caffeine induced the release of IL-6 from human myotubular cells, and its maximum release occurred 4 to 6 h after the addition of caffeine (6). Similarly, incubation of isolated rat soleus muscle with ionomycin for 60 min in the incubation media increased protein levels of IL-6 of (40). A possible explanation of the discrepancy among these previous studies regarding the secretion of IL-6 from skeletal muscle could be owing to differences in fiber type (glycolytic vs. oxidative) or animal species (rats vs. mice) used in each study. Indeed, it has been known that rat soleus muscle contains more oxidative fibers than mouse soleus muscle (41). As IL-6 secretion from the soleus muscle of rats is greater than that from the soleus muscle of mice, differences in skeletal muscle fiber types in each animal species may explain the differences in responsiveness of IL-6 secretion to an increase in intracellular calcium by caffeine and other Ca^2+^ ionophores.

On the other hand, it has been reported that intravenous acute AICAR stimulation (within 100 min) decreases the number of IL-6-vesicles in mouse skeletal myocytes, suggesting that AMPK activation can be involved in myokine secretion (11). In addition, incubation of cultured human myotubes with AICAR within 4 to 24 h increases levels of IL-6 mRNA (42). These results suggest that AMPK signaling, one of the mechanisms of the ergogenic effects of caffeine reported to date, may regulate myokine expression and release.

### Caffeine could regulate the secretion of brain-derived neurotrophic factor as a myokine

Exercise increases circulating and muscle brain-derived neurotrophic factor (BDNF), which is a myokine, in humans and mice (43). BDNF promotes fatty acid oxidation and mitochondrial biogenesis in cultured skeletal muscle cells and the skeletal muscle of mice (43–45). We also found that blood BDNF levels in healthy subjects and patients with heart failure (HF) are closely positively correlated with whole-body exercise capacity (peak oxygen uptake) by univariate analysis and was identified as independent determinants of peak oxygen uptake by multivariate analysis (46). The administration of recombinant human BDNF (rhBDNF), as well as exercise training, improved whole-body exercise performance in normal mice (45).

BDNF has been shown to be secreted by the electrical stimulation of skeletal muscle (43). However, whether BDNF secretion from skeletal muscle is regulated by caffeine or ionomycin remains unknown. The central effects of caffeine are thought to depend on the release of various neurotransmitters by the inhibition of the adenosine receptors A1 and A2a (37). Caffeine also has the effect of reducing skeletal muscle pain during exercise, which is thought to be associated with its inhibition of adenosine receptor A1 (37). Thus, the central effects of caffeine and its effects on skeletal muscle are considered to be similar. It is known that the addition of caffeine increases BDNF secretion in cultured hippocampal neurons, which is due to the increase in [Ca^2+^]i *via* the ryanodine receptor (47). It has not yet been clarified whether caffeine induces BDNF secretion in skeletal muscle cells. However, given the similarities between the central and peripheral effects of caffeine, BDNF could secrete as a myokine *via* the caffeine-induced increase in [Ca^2+^]i.

### Caffeine could regulate the secretion of musclin as a myokine

Musclin is expressed specifically in skeletal muscle (29), and is considered to be a myokine because exercise increases skeletal muscle levels of musclin protein and mRNA, and circulating levels of musclin (8). The genetic disruption of musclin causes a decrease in physical endurance and mitochondrial content, including the signaling of mitochondrial biogenesis (8). In contrast, skeletal muscle-specific musclin overexpression using adeno-associated virus 6 also increases circulating musclin (29). This suggests that skeletal muscle musclin can be secreted into the circulation. On the other hand, mRNA expression levels of musclin have also been reported to increase in a [Ca^2+^]i (addition of an ionophore and calcium itself)-dependent manner in cultured murine and human primary myoblasts (8). Although it has not yet been confirmed, caffeine administration could have the potential to induce musclin secretion by increasing the expression level of musclin.

### Activation of AMPK regulates IL-15 as a myokine

IL-15 is predominantly expressed in skeletal muscle (12), and is considered to be a myokine because exercise increases skeletal muscle levels of both IL-15 protein and mRNA, and circulating levels of the IL-15 protein (12, 48). Similarly, AICAR, an activator of AMPK, was found to increase skeletal muscle IL-15 mRNA levels in mice (12). Moreover, it is well known that IL-15 promotes fatty acid and glucose oxidation, and mitochondrial oxidative function with supercomplex formation of the electron transport chain in muscle tissue (49–53). IL-15 also inhibit skeletal muscle degradation and muscle nuclear apoptosis (54–56), and increase muscle grip strength (12). Moreover, circulating IL-15 and skeletal muscle IL-15Ra expression correlated with protein synthesis after resistance exercise (57). Furthermore, mice overexpressing IL-15 in skeletal muscle on a low-fat/low-energy diet and a high-fat/high-energy diet had increased lean body mass, including skeletal muscle (58). As described above, IL-15 is considered to be a myokine that has a generally positive effect on skeletal muscle, but whether its expression and secretion can be regulated by caffeine is a subject for future research.

## Association of myokines with acute and chronic effects of caffeine stimulation *in vitro*

The effects of exercise can be acute or chronic (1, 59). Similarly, the effects of acute and chronic muscle contraction are different (10), but whether there is a difference in myokine secretion is unclear. In this paper, we hypothesized and discussed that the ergogenic effects of caffeine are mediated by myokine secretion. Long-term (i.e., chronic) as well as single, short-term (i.e., acute) administration of caffeine produces ergogenic effects *via* intracellular calcium increases and AMPK signaling (Figure 1) (10). This is thought to mimic the effects of acute and chronic exercise (1, 59). From the viewpoint of intracellular calcium increase and AMPK signaling, myokine secretion is thought to play an important role in the effects of caffeine. However, at present, IL-6 is the only myokine that has been shown to be directly secreted from skeletal muscle upon short-term caffeine stimulation (6). Therefore, comprehensive investigation of the types of myokines that are secreted by caffeine stimulation is an important research topic. In addition, research on myokine secretion by chronic caffeine stimulation is also an unresolved issue. It is well known that chronic caffeine administration to skeletal muscle enhances glucose and lipid oxidation, and mitochondrial biogenesis, which can be explained by the effects of BDNF (44, 45).

## Indirect effects of myokines on other organs

**Figure 2:**
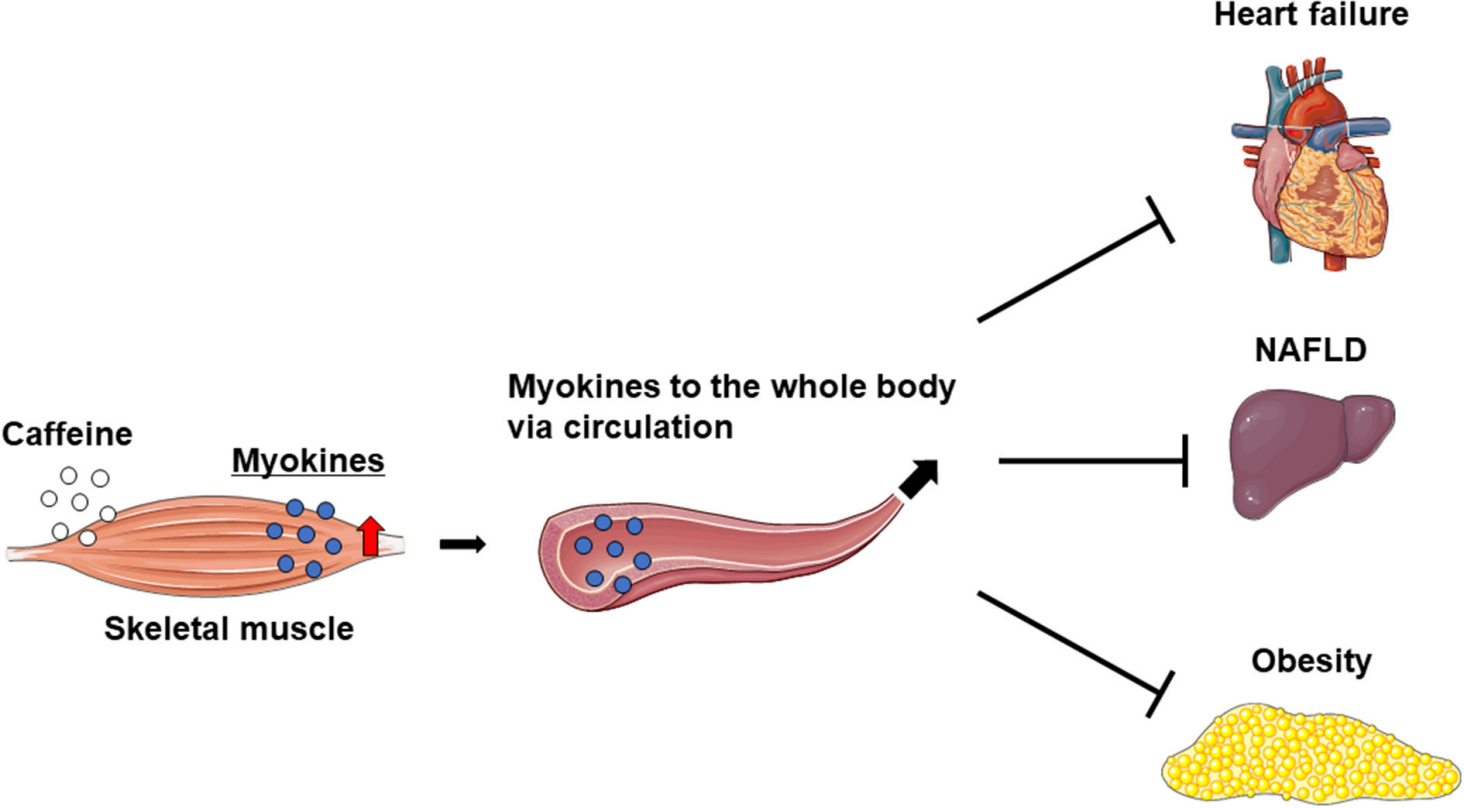
Increased myokines in skeletal muscle may improve heart failure, NAFLD, and obesity.

Caffeine intake markedly increases IL-6 levels in the skeletal muscle and blood, but not in the liver of mice. Furthermore, caffeine-stimulated skeletal muscle IL-6 production alleviated nonalcoholic fatty liver disease (NAFLD) in a rodent model (17). On the other hand, the overexpression of musclin in skeletal muscle was found to attenuate left ventricular dysfunction and myocardial fibrosis in mice with HF induced by long-term pressure overload (29). These results suggest that caffeine ameliorates myocardial remodeling *via* inducing crosstalk between the muscle and liver or heart. On the other hand, mice overexpressing IL-15 in skeletal muscle have reduced fat mass and show anti-obesity effects (52, 58). Therefore, increased myokine levels in the skeletal muscle and circulation owing to exercise and/or the intake of caffeine as an ergogenic aid may prevent or improve specific pathologies, such as myocardial remodeling in HF, NAFLD, and obesity (Figure 2).

## Future directions and perspectives

Why do the health benefits of exercise extend beyond the skeletal muscles to the whole body? Although the full mechanism still remains unclear (1), the discovery of molecules that are the key to the systemic effects of exercise, namely myokines, has greatly advanced the field of exercise physiology (3, 28). Through the discovery of myokines, which are specific molecules that have physiological activity and can be secreted into the blood, it has been shown that exercise and caffeine, an exercise mimetic, have effects not only on skeletal muscle itself, but also on remote organs *via* skeletal muscle (4, 29, 60). In addition, many mechanisms underlying the association between intracellular calcium dynamics and intracellular transport and secretion resulting from caffeine stimulation have been elucidated (26, 27), and because intracellular calcium also demonstrates characteristic dynamics during muscle contraction, it can be speculated that myokine secretion into the blood is regulated by exercise (5, 28).

However, it should be reiterated that the intracellular events reproduced by caffeine stimulation reflect some mechanisms of some modes of exercise within the larger framework of exercise. Indeed, it has been reported that the composition of proteins in the blood changes with the intensity and type of exercise performed (61), and hence attention should be paid to what type of exercise is reproduced by caffeine. Caffeine has the potential to increase our understanding of exercise-induced myokine secretion and its systemic effects, which is an interdisciplinary field between exercise physiology and cell biology. Furthermore, clarifying the mechanism of myokine secretion induced by exercise may help to resolve the effects of physical inactivity in older people and enhance the efficacy of post-injury rehabilitation. This is because some myokines have already been found to be clinically significant (46, 62, 63). In addition, not surprisingly, patients facing clinical challenges often have difficulty exercising on their own, so the systemic effects of myokines induced by caffeine stimulation as an exercise mimetic have great promise (4, 64).

In this review, we connected “(1) the positive effects of myokines on skeletal muscle” with “(2) the secretion of these myokines by caffeine and their effects on skeletal muscle” to form the hypotheses shown in Figure 1. The results of (1) and (2) are from separate studies, and it hence remains unclear whether caffeine has acute or chronic effects on skeletal muscle *via* myokines, and whether caffeine improves exercise performance.

## Conclusion

The ergogenic effects of caffeine are mediated through myokine regulation. Clarifying the underlying mechanisms will require elucidation of not only the ergogenic effects of caffeine, but also the mechanisms of the effects of exercise and myokines.

## Author contributions

All authors listed have made a substantial, direct, and intellectual contribution to the work and approved it for publication.

## Funding

This work was supported in part by Grants-in-Aid for Scientific Research (Grant Nos. JP17H04758 to ST and 21H03360 to SK) and Grants-in-Aid for Challenging Exploratory Research (Grant No. 19K22791 to ST) from the Japan Society for the Promotion of Science, grants from the Akiyama Life Science Foundation (to ST), the Suhara Memorial Foundation (to ST), and the Japan Foundation for Applied Enzymology (to ST).

## Conflict of interest

The authors declare that the research was conducted in the absence of any commercial or financial relationships that could be construed as a potential conflict of interest.

## Publisher's note

All claims expressed in this article are solely those of the authors and do not necessarily represent those of their affiliated organizations, or those of the publisher, the editors and the reviewers. Any product that may be evaluated in this article, or claim that may be made by its manufacturer, is not guaranteed or endorsed by the publisher.

## References

[1] Egan B Zierath JR. Exercise metabolism and the molecular regulation of skeletal muscle adaptation. Cell Metab. (2013) 17:162–84. 10.1016/j.cmet.2012.12.012 23395166

[2] Thyfault JP Bergouignan A. Exercise and metabolic health: beyond skeletal muscle. Diabetologia. (2020) 63:1464–74. 10.1007/s00125-020-05177-6 32529412 PMC7377236

[3] Bay ML Pedersen BK. Muscle-organ crosstalk: focus on immunometabolism. Front Physiol. (2020) 11:567881. 10.3389/fphys.2020.567881 33013484 PMC7509178

[4] Takada S Sabe H Kinugawa S. Abnormalities of skeletal muscle, adipocyte tissue, and lipid metabolism in heart failure: practical therapeutic targets. Front Cardiovasc Med. (2020) 7:79. 10.3389/fcvm.2020.00079 32478098 PMC7235191

[5] Pedersen BK Febbraio MA. Muscle as an endocrine organ: focus on muscle-derived interleukin-6. Physiol Rev. (2008) 88:1379–406. 10.1152/physrev.90100.2007 18923185

[6] Ducreux S Zorzato F Muller C Sewry C Muntoni F Quinlivan R. Effect of ryanodine receptor mutations on interleukin-6 release and intracellular calcium homeostasis in human myotubes from malignant hyperthermia-susceptible individuals and patients affected by central core disease. J Biol Chem. (2004) 279:43838–46. 10.1074/jbc.M403612200 15299003

[7] Seldin MM Peterson JM Byerly MS Wei Z Wong GW. Myonectin (CTRP15), a novel myokine that links skeletal muscle to systemic lipid homeostasis. J Biol Chem. (2012) 287:11968–80. 10.1074/jbc.M111.336834 22351773 PMC3320944

[8] Subbotina E Sierra A Zhu Z Gao Z Koganti SR Reyes S. Musclin is an activity-stimulated myokine that enhances physical endurance. Proc Natl Acad Sci U S A. (2015) 112:16042–7. 10.1073/pnas.1514250112 26668395 PMC4702977

[9] Furuichi Y Manabe Y Takagi M Aoki M Fujii NL. Evidence for acute contraction-induced myokine secretion by C2C12 myotubes. PLoS ONE. (2018) 13:e0206146. 10.1371/journal.pone.0206146 30356272 PMC6200277

[10] Carter S Solomon TPJ. *In vitro* experimental models for examining the skeletal muscle cell biology of exercise: the possibilities, challenges and future developments. Pflugers Arch. (2019) 471:413–29. 10.1007/s00424-018-2210-4 30291430

[11] Lauritzen HP Brandauer J Schjerling P Koh HJ Treebak JT Hirshman MF. Contraction and AICAR stimulate IL-6 vesicle depletion from skeletal muscle fibers *in vivo*. Diabetes. (2013) 62:3081–92. 10.2337/db12-1261 23761105 PMC3749330

[12] Crane JD Macneil LG Lally JS Ford RJ Bujak AL Brar IK. Exercise-stimulated interleukin-15 is controlled by AMPK and regulates skin metabolism and aging. Aging Cell. (2015) 14:625–34. 10.1111/acel.12341 25902870 PMC4531076

[13] Davis JK Green JM. Caffeine and anaerobic performance: ergogenic value and mechanisms of action. Sports Med. (2009) 39:813–32. 10.2165/11317770-000000000-00000 19757860

[14] Grgic J Grgic I Pickering C Schoenfeld BJ Bishop DJ Pedisic Z. Wake up and smell the coffee: caffeine supplementation and exercise performance-an umbrella review of 21 published meta-analyses. Br J Sports Med. (2020) 54:681–8. 10.1136/bjsports-2018-100278 30926628

[15] Martins GL Guilherme J Ferreira LHB De Souza-Junior TP Lancha AHJr. Caffeine and exercise performance: possible directions for definitive findings. Front Sports Act Living. (2020) 2:574854. 10.3389/fspor.2020.574854 33345139 PMC7739593

[16] Kreutzer A Graybeal AJ Moss K Braun-Trocchio R Shah M. Caffeine supplementation strategies among endurance athletes. Front Sports Act Living. (2022) 4:821750. 10.3389/fspor.2022.821750 35463835 PMC9030507

[17] Fang C Cai X Hayashi S Hao S Sakiyama H Wang X. Caffeine-stimulated muscle IL-6 mediates alleviation of non-alcoholic fatty liver disease. Biochim Biophys Acta Mol Cell Biol Lipids. (2019) 1864:271–80. 10.1016/j.bbalip.2018.12.003 30553055

[18] Mcconell GK Ng GP Phillips M Ruan Z Macaulay SL Wadley GD. Central role of nitric oxide synthase in AICAR and caffeine-induced mitochondrial biogenesis in L6 myocytes. J Appl Physiol. (2010) 108:589–95. 10.1152/japplphysiol.00377.2009 20044477

[19] Lally JSV Jain SS Han XX Snook LA Glatz JFC Luiken J. Caffeine-stimulated fatty acid oxidation is blunted in CD36 null mice. Acta Physiol. (2012) 205:71–81. 10.1111/j.1748-1716.2012.02396.x 22463611

[20] Moore TM Mortensen XM Ashby CK Harris AM Kump KJ Laird DW. The effect of caffeine on skeletal muscle anabolic signaling and hypertrophy. Appl Physiol Nutr Metab. (2017) 42:621–9. 10.1139/apnm-2016-0547 28177708

[21] Talanian JL Spriet LL. Low and moderate doses of caffeine late in exercise improve performance in trained cyclists. Appl Physiol Nutr Metab. (2016) 41:850–5. 10.1139/apnm-2016-0053 27426699

[22] Ramirez-Maldonado M Jurado-Fasoli L Del Coso J Ruiz JR Amaro-Gahete FJ. Caffeine increases maximal fat oxidation during a graded exercise test: is there a diurnal variation? J Int Soc Sports Nutr. (2021) 18:5. 10.1186/s12970-020-00400-6 33413459 PMC7792284

[23] Endo M. Calcium release from the sarcoplasmic reticulum. Physiol Rev. (1977) 57:71–108. 10.1152/physrev.1977.57.1.71 13441

[24] Endo M. Calcium-induced calcium release in skeletal muscle. Physiol Rev. (2009) 89:1153–76. 10.1152/physrev.00040.2008 19789379

[25] Maurya SK Herrera JL Sahoo SK Reis FCG Vega RB Kelly DP. Sarcolipin signaling promotes mitochondrial biogenesis and oxidative metabolism in skeletal muscle. Cell Rep. (2018) 24:2919–31. 10.1016/j.celrep.2018.08.036 30208317 PMC6481681

[26] Olson EN Williams RS. Calcineurin signaling and muscle remodeling. Cell. (2000) 101:689–92. 10.1016/S0092-8674(00)80880-6 10892739

[27] Stojilkovic SS. Ca2+-regulated exocytosis and SNARE function. Trends Endocrinol Metab. (2005) 16:81–3. 10.1016/j.tem.2005.02.002 15808803

[28] Pedersen BK Febbraio MA. Muscles, exercise and obesity: skeletal muscle as a secretory organ. Nat Rev Endocrinol. (2012) 8:457–65. 10.1038/nrendo.2012.49 22473333

[29] Szaroszyk M Kattih B Martin-Garrido A Trogisch FA Dittrich GM Grund A. Skeletal muscle derived Musclin protects the heart during pathological overload. Nat Commun. (2022) 13:149. 10.1038/s41467-021-27634-5 35013221 PMC8748430

[30] Egawa T Hamada T Ma X Karaike K Kameda N Masuda S. Caffeine activates preferentially alpha1-isoform of 5'AMP-activated protein kinase in rat skeletal muscle. Acta Physiol. (2011) 201:227–38. 10.1111/j.1748-1716.2010.02169.x 21241457

[31] Mathew TS Ferris RK Downs RM Kinsey ST Baumgarner BL. Caffeine promotes autophagy in skeletal muscle cells by increasing the calcium-dependent activation of AMP-activated protein kinase. Biochem Biophys Res Commun. (2014) 453:411–8. 10.1016/j.bbrc.2014.09.094 25268764 PMC8428546

[32] Allen DL Uyenishi JJ Cleary AS Mehan RS Lindsay SF Reed JM. Calcineurin activates interleukin-6 transcription in mouse skeletal muscle in vivo and in C2C12 myotubes in vitro. Am J Physiol Regul Integr Comp Physiol. (2010) 298:R198–210. 10.1152/ajpregu.00325.2009 19907005 PMC2806210

[33] Petersen EW Carey AL Sacchetti M Steinberg GR Macaulay SL Febbraio MA. Acute IL-6 treatment increases fatty acid turnover in elderly humans *in vivo* and in tissue culture *in vitro*. Am J Physiol Endocrinol Metab. (2005) 288:E155–162. 10.1152/ajpendo.00257.2004 15383370

[34] Carey AL Steinberg GR Macaulay SL Thomas WG Holmes AG Ramm G. Interleukin-6 increases insulin-stimulated glucose disposal in humans and glucose uptake and fatty acid oxidation in vitro via AMP-activated protein kinase. Diabetes. (2006) 55:2688–97. 10.2337/db05-1404 17003332

[35] Glund S Deshmukh A Long YC Moller T Koistinen HA Caidahl K. Interleukin-6 directly increases glucose metabolism in resting human skeletal muscle. Diabetes. (2007) 56:1630–7. 10.2337/db06-1733 17363741

[36] Serrano AL Baeza-Raja B Perdiguero E Jardi M Munoz-Canoves P. Interleukin-6 is an essential regulator of satellite cell-mediated skeletal muscle hypertrophy. Cell Metab. (2008) 7:33–44. 10.1016/j.cmet.2007.11.011 18177723

[37] Mclellan TM Caldwell JA Lieberman HR. A review of caffeine's effects on cognitive, physical and occupational performance. Neurosci Biobehav Rev. (2016) 71:294–312. 10.1016/j.neubiorev.2016.09.001 27612937

[38] Tallis J Duncan MJ James RS. What can isolated skeletal muscle experiments tell us about the effects of caffeine on exercise performance? Br J Pharmacol. (2015) 172:3703–13. 10.1111/bph.13187 25988508 PMC4523329

[39] Glund S Treebak JT Long YC Barres R Viollet B Wojtaszewski JF. Role of adenosine 5'-monophosphate-activated protein kinase in interleukin-6 release from isolated mouse skeletal muscle. Endocrinology. (2009) 150:600–6. 10.1210/en.2008-1204 18818284

[40] Holmes AG Watt MJ Carey AL Febbraio MA. Ionomycin, but not physiologic doses of epinephrine, stimulates skeletal muscle interleukin-6 mRNA expression and protein release. Metabolism. (2004) 53:1492–5. 10.1016/j.metabol.2004.05.015 15536607

[41] Gregorevic P Meznarich NA Blankinship MJ Crawford RW Chamberlain JS. Fluorophore-labeled myosin-specific antibodies simplify muscle-fiber phenotyping. Muscle Nerve. (2008) 37:104–6. 10.1002/mus.20877 17691104 PMC2788960

[42] Weigert C Dufer M Simon P Debre E Runge H Brodbeck K. Upregulation of IL-6 mRNA by IL-6 in skeletal muscle cells: role of IL-6 mRNA stabilization and Ca2+-dependent mechanisms. Am J Physiol Cell Physiol. (2007) 293:C1139–1147. 10.1152/ajpcell.00142.2007 17615159

[43] Matthews VB Astrom MB Chan MH Bruce CR Krabbe KS Prelovsek O. Brain-derived neurotrophic factor is produced by skeletal muscle cells in response to contraction and enhances fat oxidation via activation of AMP-activated protein kinase. Diabetologia. (2009) 52:1409–18. 10.1007/s00125-009-1364-1 19387610

[44] Matsumoto J Takada S Kinugawa S Furihata T Nambu H Kakutani N. Brain-derived neurotrophic factor improves limited exercise capacity in mice with heart failure. Circulation. (2018) 138:2064–6. 10.1161/CIRCULATIONAHA.118.035212 30372141

[45] Matsumoto J Takada S Furihata T Nambu H Kakutani N Maekawa S. Brain-Derived neurotrophic factor improves impaired fatty acid oxidation *via* the activation of adenosine monophosphate-activated protein kinase-a - proliferator-activated receptor-r coactivator-1a signaling in skeletal muscle of mice with heart failure. Circ Heart Fail. (2021) 14:e005890. 10.1161/CIRCHEARTFAILURE.119.005890 33356364

[46] Fukushima A Kinugawa S Homma T Masaki Y Furihata T Yokota T. Decreased serum brain-derived neurotrophic factor levels are correlated with exercise intolerance in patients with heart failure. Int J Cardiol. (2013) 168:e142–144. 10.1016/j.ijcard.2013.08.073 24029660

[47] Lao-Peregrin C Ballesteros JJ Fernandez M Zamora-Moratalla A Saavedra A Gomez Lazaro M. Caffeine-mediated BDNF release regulates long-term synaptic plasticity through activation of IRS2 signaling. Addict Biol. (2017) 22:1706–18. 10.1111/adb.12433 27457910 PMC5697621

[48] Yang H Chang J Chen W Zhao L Qu B Tang C. Treadmill exercise promotes interleukin 15 expression in skeletal muscle and interleukin 15 receptor alpha expression in adipose tissue of high-fat diet rats. Endocrine. (2013) 43:579–85. 10.1007/s12020-012-9809-6 23076740

[49] Almendro V Busquets S Ametller E Carbo N Figueras M Fuster G. Effects of interleukin-15 on lipid oxidation: disposal of an oral [(14)C]-triolein load. Biochim Biophys Acta. (2006) 1761:37–42. 10.1016/j.bbalip.2005.12.006 16458591

[50] Busquets S Figueras M Almendro V Lopez-Soriano FJ Argiles JM. Interleukin-15 increases glucose uptake in skeletal muscle. An antidiabetogenic effect of the cytokine. Biochim Biophys Acta. (2006) 1760:1613–7. 10.1016/j.bbagen.2006.09.001 17056184

[51] Krolopp JE Thornton SM Abbott MJ. IL-15 Activates the Jak3/STAT3 signaling pathway to mediate glucose uptake in skeletal muscle cells. Front Physiol. (2016) 7:626. 10.3389/fphys.2016.00626 28066259 PMC5167732

[52] Nadeau L Aguer C. Interleukin-15 as a myokine: mechanistic insight into its effect on skeletal muscle metabolism. Appl Physiol Nutr Metab. (2019) 44:229–38. 10.1139/apnm-2018-0022 30189147

[53] Nadeau L Patten DA Caron A Garneau L Pinault-Masson E Foretz M. IL-15 improves skeletal muscle oxidative metabolism and glucose uptake in association with increased respiratory chain supercomplex formation and AMPK pathway activation. Biochim Biophys Acta Gen Subj. (2019) 1863:395–407. 10.1016/j.bbagen.2018.10.021 30448294 PMC6310627

[54] Carbo N Lopez-Soriano J Costelli P Busquets S Alvarez B Baccino FM. Interleukin-15 antagonizes muscle protein waste in tumour-bearing rats. Br J Cancer. (2000) 83:526–31. 10.1054/bjoc.2000.1299 10945502 PMC2374658

[55] Quinn LS Anderson BG Drivdahl RH Alvarez B Argiles JM. Overexpression of interleukin-15 induces skeletal muscle hypertrophy *in vitro*: implications for treatment of muscle wasting disorders. Exp Cell Res. (2002) 280:55–63. 10.1006/excr.2002.5624 12372339

[56] Busquets S Figueras MT Meijsing S Carbo N Quinn LS Almendro V. Interleukin-15 decreases proteolysis in skeletal muscle: a direct effect. Int J Mol Med. (2005) 16:471–6. 10.3892/ijmm.16.3.471 16077957

[57] Perez-Lopez A Mckendry J Martin-Rincon M Morales-Alamo D Perez-Kohler B Valades D. Skeletal muscle IL-15/IL-15Ralpha and myofibrillar protein synthesis after resistance exercise. Scand J Med Sci Sports. (2018) 28:116–25. 10.1111/sms.12901 28449327

[58] Quinn LS Anderson BG Strait-Bodey L Stroud AM Argiles JM. Oversecretion of interleukin-15 from skeletal muscle reduces adiposity. Am J Physiol Endocrinol Metab. (2009) 296:E191–202. 10.1152/ajpendo.90506.2008 19001550 PMC2636988

[59] Chow LS Gerszten RE Taylor JM Pedersen BK Van Praag H Trappe S. Exerkines in health, resilience and disease. Nat Rev Endocrinol. (2022) 18:273–89. 10.1038/s41574-022-00641-2 35304603 PMC9554896

[60] Gubert C Hannan AJ. Exercise mimetics: harnessing the therapeutic effects of physical activity. Nat Rev Drug Discov. (2021) 20:862–79. 10.1038/s41573-021-00217-1 34103713

[61] Morville T Sahl RE Moritz T Helge JW Clemmensen C. Plasma metabolome profiling of resistance exercise and endurance exercise in humans. Cell Rep. (2020) 33:108554. 10.1016/j.celrep.2020.108554 33378671

[62] Fukushima A Kinugawa S Homma T Masaki Y Furihata T Yokota T. Serum brain-derived neurotropic factor level predicts adverse clinical outcomes in patients with heart failure. J Card Fail. (2015) 21:300–6. 10.1016/j.cardfail.2015.01.003 25639689

[63] Nakano I Kinugawa S Hori H Fukushima A Yokota T Takada S. Serum brain-derived neurotrophic factor levels are associated with skeletal muscle function but not with muscle mass in patients with heart failure. Int Heart J. (2020) 61:96–102. 10.1536/ihj.19-400 31956152

[64] Takada S Sabe H Kinugawa S. Treatments for skeletal muscle abnormalities in heart failure: sodium-glucose transporter 2 and ketone bodies. Am J Physiol Heart Circ Physiol. (2022) 322:H117–28. 10.1152/ajpheart.00100.2021 34860594

